# TiO_2_-catalyzed synthesis of sugars from formaldehyde in extraterrestrial impacts on the early Earth

**DOI:** 10.1038/srep23199

**Published:** 2016-03-16

**Authors:** Svatopluk Civiš, Rafał Szabla, Bartłomiej M. Szyja, Daniel Smykowski, Ondřej Ivanek, Antonín Knížek, Petr Kubelík, Jiří Šponer, Martin Ferus, Judit E. Šponer

**Affiliations:** 1 J. Heyrovský Institute of Physical Chemistry, Academy of Sciences of the Czech Republic, Dolejškova 3, CZ–182 23 Prague 8, Czech Republic; 2 Institute of Biophysics, Academy of Sciences of the Czech Republic, Královopolská 135, CZ–612 65 Brno, Czech Republic; 3 Inorganic Materials Chemistry, Department of Chemical Engineering and Chemistry, Eindhoven University of Technology, Den Dolech 2, 5612 AZ Eindhoven, The Netherlands; 4 Division of Fuels Chemistry and Technology, Faculty of Chemistry, Wrocław University of Technology, Gdańska 7/9, 50-344 Wrocław, Poland; 5 Faculty of Mechanical and Power Engineering, Wrocław University of Technology, Ul. Wybrzeże Wyspiańskiego 27, 50-370, Wrocław; 6 CEITEC – Central European Institute of Technology, Masaryk University, Campus Bohunice, Kamenice 5, CZ–62500 Brno, Czech Republic; 7 Institute of Physics, Czech Academy of Sciences, Na Slovance 2, 182 21 Prague 8, Czech Republic

## Abstract

Recent synthetic efforts aimed at reconstructing the beginning of life on our planet point at the plausibility of scenarios fueled by extraterrestrial energy sources. In the current work we show that beyond nucleobases the sugar components of the first informational polymers can be synthesized in this way. We demonstrate that a laser-induced high-energy chemistry combined with TiO_2_ catalysis readily produces a mixture of pentoses, among them ribose, arabinose and xylose. This chemistry might be highly relevant to the Late Heavy Bombardment period of Earth’s history about 4–3.85 billion years ago. In addition, we present an in-depth theoretical analysis of the most challenging step of the reaction pathway, i.e., the TiO_2_-catalyzed dimerization of formaldehyde leading to glycolaldehyde.

Formation of simple sugars from one carbon feedstock molecules on early Earth has for long been considered as one of the most fundamental steps in the prebiotic syntheses leading to RNA. The oligomerization of formaldehyde, known as the formose reaction^1^,^2^, is the most widely recognized scenario in this context. Nevertheless, the formose reaction is plausible only if glycolaldehyde is present in the reaction mixture. Otherwise, the initial formaldehyde dimerization to glycolaldehyde is not feasible since it requires opposite polarity on the carbon atoms of the two reacting molecules. It has been demonstrated that if glycolaldehyde is present in the reaction mixture in trace amounts, simple sugars can be produced in autocatalytic cycles in the presence of borate minerals^3^. Since glycolaldehyde has been detected in our galaxy^4^, it was suggested that glycolaldehyde necessary for the synthesis of terrestrial sugars has been delivered from the space^5^. Another alternatives for glycolaldehyde production on the primitive Earth include UV–photolysis of formaldehyde^6^ and reaction of CH_4_ with CO_2_^7^. The borate-chemistry proposed by Benner and coworkers^3^,^8^,^9^,^10^ offers not only an elegant way to the sequestration of pentoses from the prebiotic mix but also shows that the reaction can generate a sufficient amount of glycolaldehyde necessary to maintain production of aldopentoses. In this model pentoses are derived from glycolaldehyde and glyceraldehyde. The role of borates is to promote enolization of glycolaldehyde and slow down enolization of glyceraldehyde. In addition, as a side reaction branched pentoses are formed from the enol form of glyceraldehyde again in a borate-catalyzed reaction, and the retroaldol fragmentation thereof then produces sufficient amount of glycolaldehyde necessary to maintain the autocatalytic cycle. Zubay *et al.* have shown that Pb^2+^ cations can significantly increase the otherwise low yield of the formose reaction^11^. Reid and Orgel reported yields as high as 40% for the synthesis of sugars catalyzed by CaCO_3_ and apatite^12^. The peptide-catalyzed synthesis of sugars reported by Pizzarello and Weber proceeds with a relatively low yield but stereoselectively leads to D-sugars^13^.

Previously, we have successfully used Laser Induced Dielectric Breakdown (LIDB) plasma chemistry to simulate synthesis of nucleobases from small-molecular precursors in a high–density energy event, like impact of an extraterrestrial body^14^,^15^,^16^. It has been shown that such events are especially relevant to the Late Heavy Bombardment period ca. 4 billion years ago, which roughly coincides with the time when terrestrial life emerged.

The approach used in our previous papers^14^,^15^,^16^ is thus relevant to the impact of an icy, extraterrestrial body on an early Earth atmosphere. The great synthetic advantage of extraterrestrial impacts lies in their very broad energy spectrum: via a rovibrational excitation of the reaction components it facilitates a wide variety of chemical transformations. In the current work, we extend this approach towards the synthesis of sugars. We demonstrate that the crucial first step of the formose reaction yielding glycolaldehyde could be catalyzed by an anatase (TiO_2_) surface activated in such an impact.

## Results and Discussion

**Figure 1:**
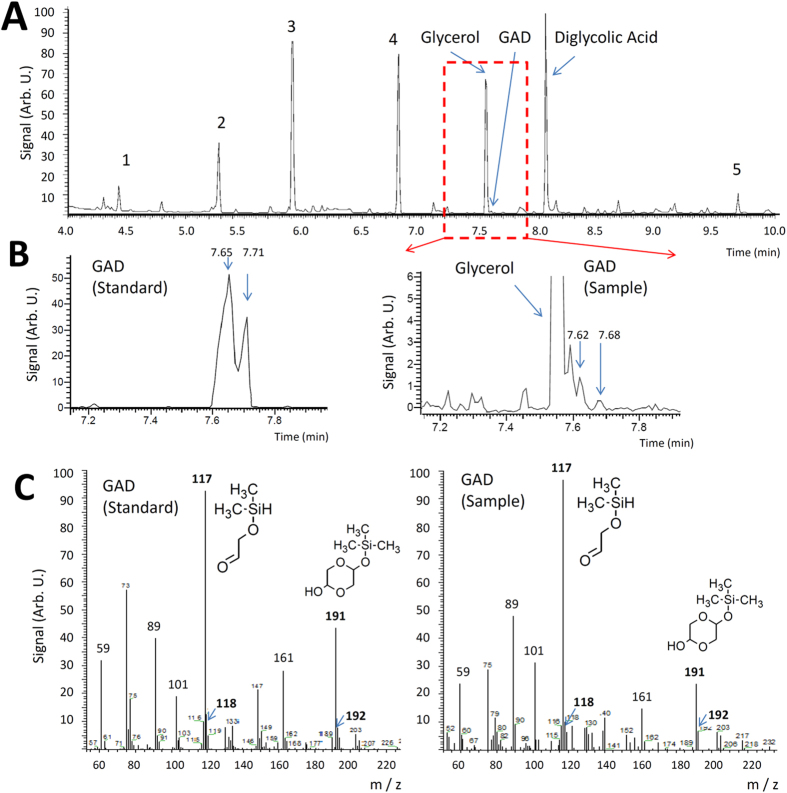
Panel (**A**) chromatogram of the derivatized sample of paraformaldehyde + TiO_2_ mixture treated with 10 laser pulses. Peaks of manifold products of mutual reactions among derivatization agents are marked 1–5. Glycerol and diglycolic acid have been identified using the NIST library^43^. Panel (**B**) chromatographic double peak of glycolaldehyde dimer (GAD) in a standard (100 ppm in deionized water) and the similar peak detected in the irradiated sample (Sample). Panel (**C**) Mass spectra of GAD in the irradiated sample as well as that of GAD standard along with two selected signatures of derivatized monomer and dimer.

**Figure 2:**
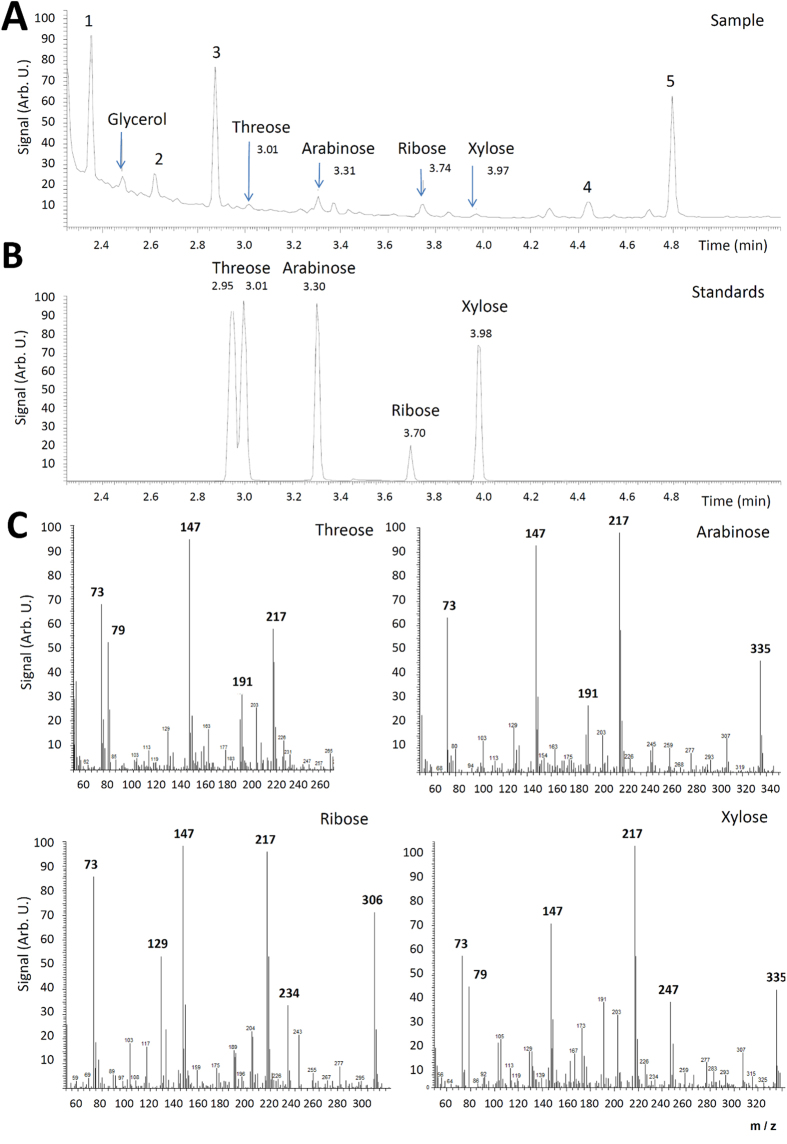
Panel (**A**) chromatogram of the derivatized sample of paraformaldehyde + TiO_2_ mixture treated with 10 laser pulses. Peaks of manifold products of mutual reactions among derivatization agents are marked 1–5. Panel (**B**) chromatogram of selected sugars measured for comparison. For the corresponding mass spectra detected in the sample see panel (**C**). For details see the Supplementary Information.

In our experiments, we mixed 1.0 g of paraformaldehyde sample with deionized water and a catalytic amount (0.1 g) of anatase form of titania TiO_2_. Then a frozen sample was prepared using liquid nitrogen, which was subsequently treated with 10 pulses of the Prague Asterix Laser System (PALS) (wavelength of 1.315 μm, energy of 150 J, mean output density of 10^15^ W/cm^2^) in a cell filled with 760 Torr of nitrogen inert gas. Formation of glycolaldehyde was hinted by an absorption at 853 cm^−1^ (assigned to a C-C stretching vibration)^17^ in the high resolution infrared spectrum of the vapor-phase products shown in Fig. S1 in the Supporting Information. An independent subsequent Gas Chromatography – Mass Spectroscopic (GC–MS) analysis after derivatization by silylation has confirmed presence of glycolaldehyde in an amount of 10 ppm in the evaporated liquid-phase product (see Fig. 1). In addition, we have identified four sugars, such as threose, arabinose, ribose and xylose, in a total amount of 110 ppm together with glycerol and diglycolic acid in the GC-MS spectra of the higher molecular weight products (see Fig. 2). In contrast, glycolaldehyde and the above mentioned sugars were either undetectable or formed in very low concentration (near the detection limit of >1 mg/kg) in the sample irradiated in the absence of TiO_2_. No sugars, glycerol, diglycolic acid or glycolaldehyde were detected in the non–irradiated blank sample prepared simultaneously (see Fig. S3 in the Supplementary Information). This shows that TiO_2_–catalysis and activation by high–energy pulses strongly cooperate in the studied scenario of sugar synthesis from formaldehyde.

Our results indicate that in an impact-chemistry sugars can be synthesized from pure formaldehyde without even trace amounts of glycolaldehyde present in the reaction mixture. This implies that glycolaldehyde is produced in the very first step of the reaction pathway, via dimerization of formaldehyde promoted by the TiO_2_-anatase surface. (Let us note here, that glycolaldehyde detected in the product mixture could also be partly formed via retroaldolization of tetroses in further stages of the reaction, see in ref. 3). We suggest that high-energy pulses activate the TiO_2_-anatase surface by increasing the number of oxygen vacancies in the crystal structure^18^,^19^.

**Figure 3:**
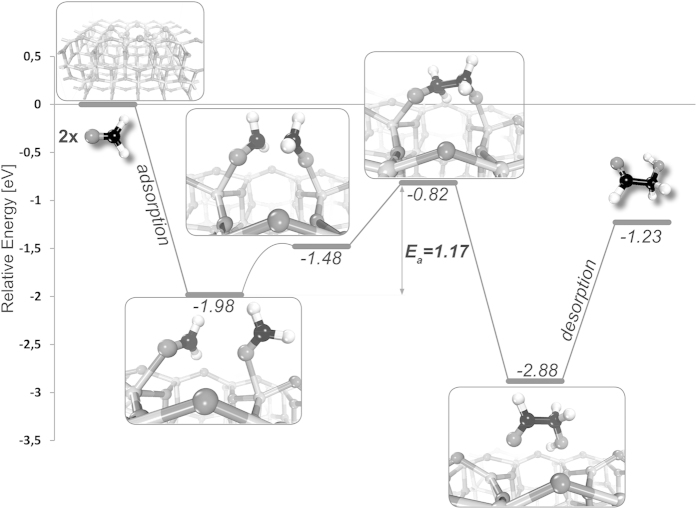
Reaction path leading to glycolaldehyde on the modified TiO_2_-anatase (001) surface. Relative energies were derived from DFT computations.

The feasibility of formaldehyde to glycolaldehyde dimerization is further supported by our quantum chemical calculations. We carried out density functional theory (DFT) computations of this reaction on the reactive (001) surface of TiO_2_-anatase with a surface O-vacancy placed in the middle of a 4 × 5 supercell. Since the O-vacancy forms a biradical state with each excess electron localized on one unsaturated Ti^3+^ cation^20^, we performed spin-polarized calculations with enforced triplet multiplicity. For this purpose we utilized the Quantum Espresso plane-wave DFT code^21^, and the PBE (Perdew-Burke-Ernzernhof) functional^22^ (see the Supplementary Information for more details). The results indicate that two formaldehyde molecules can be easily adsorbed on two neighboring unsaturated Ti^3+^ cations, with the total adsorption energy of −2.0 eV. These two formaldehydes can then effectively dimerize yielding glycolaldehyde with the energy barrier of approximately 1.2 eV (see Fig. 3 and Fig. S2 of the Supplementary Information for more detailed view of the reaction path). The biradical character of the defect enables the C-C bond formation and subsequent hydrogen atom transfer to occur within two reaction steps separated by a shallow plateau on the potential energy surface. Since the adsorption energy of the product is −1.7 eV, it is favorable for two further formaldehyde molecules to substitute glycolaldehyde in the reaction center. Therefore, the presence of O-vacancies enables a very effective catalytic cycle, without poisoning the active site of the TiO_2_ surface. Analogous computations conducted on the regular (001) surface showed that such catalyst poisoning occurs when no O-vacancies are present, i.e. the adsorption energy of the product is lower by 1.1 eV than the adsorption energy of two formaldehyde molecules. Thus, in agreement with the experimental findings, the creation of O-vacancies is essential to activate the catalyst and promote the formation of sugars. According to the theoretical results, the oxygen-deficient TiO_2_(001) surface also prevents the formation of C-O bond by anchoring oxygen atoms of the reacting formaldehyde molecules. Even though no polarity inversion is generated on any of the reacting species, glycolaldehyde formation is facilitated by oxygen-anchoring and localizing the unpaired electrons on the carbon atoms as the formaldehyde molecules approach each other.

It is reasonable to assume that the catalyst of the reaction, i.e. photoactive anatase TiO_2_, was available in a sufficiently high concentration on the early Earth. Anatase is known as a part of igneous metamorphic, weathered and hydrothermally altered rocks^23^ or meteoritic materials: see e.g. the Allan Hills meteorite A77307 (type: CO3.0 ordinary chondrite)^24^, the Martian meteorite EETA79001^25^, or the Chicxulub impact crater^26^. Our previous laboratory studies show that the catalytic activity of photoactive anatase TiO_2_ can be boosted by annealing at high temperature due to an increase of the number of vacancies inside the TiO_2_ structure^18^,^19^,^27^. Since vacancies are believed to be responsible for the catalytic activity, this suggests that the rapid rise of temperature during the extraterrestrial impact could activate TiO_2_ for catalysis. As our calculations suggest, if the TiO_2_-surface is activated, the dimerization proceeds with a relatively low activation energy in an exergonic manner.

As Benner *et al.* in ref. 9 formulate “*Electrical discharge almost certainly generated HCHO continuously in the early terran atmosphere*”^28^. This view is considered nowadays as a consensus supported by many other literature sources^7^,^29^,^30^. Moreover, formaldehyde is a rather common molecule in the space: it has been detected in extraterrestrial icy bodies, like comets^31^,^32^,^33^,^34^,^35^ and dark nebulas^36^. This suggests that formaldehyde must have been at least locally available on Earth during the Early and Late Heavy Bombardment eras either as a component of the primeval atmosphere or from extraterrestrial sources.

Thus, our experiments are compatible with a scenario, in which a high–energy impact is combined with terrestrial volcanic activity. Such an event could be plausible in a volcanic early Earth environment, when the impact of an extraterrestrial body could create the shock–wave necessary to push through the experimentally observed high–energy chemistry leading to glycolaldehyde and simple sugars.

The yield of the sugar synthesis presented above is relatively low (0.01%) as compared to other works reporting yields of 10–40%^3^,^11^,^12^. Nonetheless, one has to take into account that impact activities on the early Earth during the Late Heavy Bombardment were much higher than today, which is perhaps the best demonstrated by the fact that the amount of extraterrestrial material delivered to the Earth was astonishingly high and approached 10^9^ tons/year^16^,^37^. In this light, even these relatively low yielding processes could produce an enormous amount of carbohydrates to establish a firm chemical background for the emergence of life.

Likewise, the current results, along with our previous related work^16^, may have an extraterrestrial implication as well: they may provide a clue for the origin of sugars and nucleobases in meteoritic materials^38^,^39^. In other words, they suggest that simple organic precursors present in impactors are transformed into more complex compounds *during* the fall of an icy extraterrestrial body into a planetary atmosphere.

## Methods

### Measurement of the high–resolution infrared spectrum of irradiated samples and vapor–phase standards

Glass irradiation cell equipped with a Pyrex window of 10 cm diameter has been filled with 1 g of paraformaldehyde (reagent grade, crystalline, CAS 30525–89–4, Sigma Aldrich), 1 ml of deionized water and 0.1 g of anatase TiO_2_ (99.8%, powder, CAS 1317–70–0, Sigma Aldrich), and 1 atm of inert nitrogen gas. The sample was then frozen using liquid nitrogen, transferred to the Prague Asterix Laser System facility (PALS) and irradiated with 10 laser pulses of 150 J in energy (time interval ≈350 ps, wavelength of 1.315 μm, output of 428 MW). The laser beam has been focused using CaF_2_ lens to achieve an output density approximately of 10^14^–10^16^ W/cm^2^. The experiment mimics the high–density energy plasma in an asteroid impact (plasma temperature of 4500 K, shock wave, emission of hard UV and XUV radiation).

Prior to spectroscopic measurements the frozen samples were melted in vacuum. High resolution Fourier transform infrared (HR–FTIR) spectra of the vapor phase were measured in a multipass White cell reaching an optical path of 35 m. The cell was interfaced to a sealable glass vacuum line used for the transfer of the vapors formed upon the evaporation of the icy reaction mixture from the irradiation cell.

The spectrometer Bruker IFS 125 HR was subsequently evacuated and operated in the measurement mode from 650–5500 cm^−1^ using a HgCdTe nitrogen cooled detector and a KBr beamsplitter. 100 scans were acquired with 40 kHz scanning mirror speed with a resolution of 0.02 cm^−1^. The measured interferograms were apodised with the Blackmann–Harris apodisation function.

For comparison, we have also recorded the spectrum of a sample, which was prepared in the same way as described above, but did not contain anatase TiO_2_. To identify the products formed upon the simulated high–density energy event, we have also recorded the vapor–phase spectra of paraformaldehyde (reagent grade, crystalline, CAS 30525–89–4, Sigma Aldrich), glycolaldehyde (crystalline dimer, mixture of stereoisomers, CAS 23147–58–2, Sigma Aldrich) and glyceraldehyde (DL mixture, assay ≥90%, CAS 56–82–6 Sigma Aldrich) – water mixtures. 1 g of powdered samples has been mixed with deionized water in a vessel, subsequently frozen by liquid nitrogen and evacuated. The frozen samples have been melted again and they have been evaporated under continuous stream of inert nitrogen gas (5 Torr) to the multipass cell in the temperature range from 50 °C up to 130 °C. 100 scans were recorded to acquire the spectra during the evaporation procedure.

### GC–MS analysis of the non–volatile fraction of the products formed upon irradiation with a high–power laser

The irradiated and melted samples were evaporated under vacuum inside a vial vessel and analyzed for the presence of saccharides. The measurements were performed using a ITQ 1100 GC–Ion Trap MS system (ThermoScientific, USA), equipped with an Xcalibur MS Platform using a non–polar TG–SQC column (ThermoScientific, USA). 17 μL of hexamethyldisilazane (99% HMDS, CAS 999–97–3, Sigma Aldrich), 6 μL of chlorotrimethylsilane (99% TMCS, CAS 75–77–4, Sigma Aldrich), and 52 μL of pyridine (99.5% anhydrous, Scharlau) were added to the residue as derivatization agents and aprotic solvent, respectively. The vial was then heated at 70 °C for two hours. Subsequently, 0.5 μL of the sample was injected into the chromatograph, and the measurements were performed using a column temperature range of 180–280 °C with a temperature gradient of 30 °C min^−1^. The mass spectrum was compared with the GC chromatograms and MS spectra of D–forms of ribose, lyxose, xylose (99%), arabinose (98%), threose (60% syrup), ribulose (1 M solution) and xylulose and xylose (98% syrup) standards (all from Sigma Aldrich). Liquid–phase standards (i.e. threose, ribulose and xylulose) were evaporated under vacuum in the presence of phosphorus pentoxide prior to GC–MS analysis.

### Quantum Chemical Calculations

Density Functional Theory calculations were performed using the Perdew-Burke-Ernzerhof^22^ exchange and correlation functional (abbreviated as PBE) within the generalized gradient approximation (GGA). The PBE functional was shown to yield generally very reliable results for anatase. In the calculations we considered a 4 × 5 (15.104 Å × 18.880 Å) surface supercell and a vacuum slab of 19.0 Å to separate the periodic images along the direction of the surface normal. We utilized the spin-restricted formalism when no surface defects were taken into consideration. In the case of surfaces containing O-vacancies we performed spin-polarized calculations with fixed triplet multiplicity, since it was shown that removal of a single oxygen atom from the anatase crystal results in the formation of a biradical in the electronic ground state^20^. The wave functions were expanded in plane waves applying the kinetic energy cutoff of 50 Ry, whereas a cutoff of 200 Ry was used for the augmented density. The minimum energy path leading to the surface-assisted glycolaldehyde formation was obtained with the Nudged Elastic Band method^40^. During this procedure 16 intermediate images were optimized on the path leading from the adsorbed substrates to the products. Additional single point computations with the HSE06 range-separated functional^41^ were performed on the PBE-optimized geometries to test the reliability of the generalized gradient approximation in estimating the relative energy differences. In the utilized HSE06 functional 25% of exact Hartree-Fock exchange was mixed with 75% of the PBE exchange in the short-range part, whereas the long-range part of the exchange potential was essentially described by PBE terms. The corresponding kinetic energy cutoff for the exact exchange operator calculations was set to 50 Ry. Electron-core interactions in both the PBE and HSE06 computations were described using the Troullier-Martins norm-conserving pseudopotentials^42^. The QUANTUM ESPRESSO package was used for all computations described above^21^.

## Additional Information

**How to cite this article**: Civiš, S. *et al.* TiO_2_-catalyzed synthesis of sugars from formaldehyde in extraterrestrial impacts on the early Earth. *Sci. Rep.* **6**, 23199; doi: 10.1038/srep23199 (2016).

## Supplementary Material

Supplementary Information
