# Corrigendum: TiO_2_-catalyzed synthesis of sugars from formaldehyde in extraterrestrial impacts on the early Earth

**DOI:** 10.1038/srep27962

**Published:** 2016-06-20

**Authors:** Svatopluk Civiš, Rafał Szabla, Bartłomiej M. Szyja, Daniel Smykowski, Ondřej Ivanek, Antonín Knížek, Petr Kubelík, Jiří Šponer, Martin Ferus, Judit E. Šponer

Scientific Reports 6: Article number: 2319910.1038/srep23199; published online:03 16 2016; updated: 06 20 2016

The Acknowledgements section in this Article is incomplete.

“We thank professor Rutger van Santen for helpful discussions. This research has been financially supported by the Ministry of Education, Youth and Sports of the Czech Republic under the project CEITEC 2020 (LQ1601). The authors would like also to thank the PALS facility for supporting the experiments, particularly Dr. Jiří Skála, Ing. Jiří Ullschmied, Pavel Prchal, and Jakub Mareš. We also thank the Ministry of Education, Youth and Sports of the Czech Republic for supporting the PALS infrastructure operation in the framework of the project LM 2010014. The calculations have been partially carried out on the Cartesius supercomputer at SURFsara, within the project SH-203-14. B.Sz and D.S. would like to acknowledge the support by a statutory activity subsidy from the Polish Ministry of Science and Higher Education for the Faculty of Chemistry and for the Faculty of Mechanical and Power Engineering of Wroclaw University of Technology for year 2016. B.Sz. and D.S. would like to acknowledge the Interdisciplinary Centre for Mathematical and Computational Modeling (ICM) and Wroclaw Center of Networking and Supercomputing (WCSS) for providing the access to supercomputer facilities (grant nr. G56-4).”

should read:

“We thank professor Rutger van Santen for helpful discussions. This research has been financially supported by the Ministry of Education, Youth and Sports of the Czech Republic under the project CEITEC 2020 (LQ1601) and by the Grant Agency of the Czech Republic, grant number 14-12010S. The authors would like also to thank the PALS facility for supporting the experiments, particularly Dr. Jiří Skála, Ing. Jiří Ullschmied, Pavel Prchal, and Jakub Mareš. We also thank the Ministry of Education, Youth and Sports of the Czech Republic for supporting the PALS infrastructure operation in the framework of the project LM 2010014. The calculations have been partially carried out on the Cartesius supercomputer at SURFsara, within the project SH-203-14. B.Sz and D.S. would like to acknowledge the support by a statutory activity subsidy from the Polish Ministry of Science and Higher Education for the Faculty of Chemistry and for the Faculty of Mechanical and Power Engineering of Wroclaw University of Technology for year 2016. B.Sz. and D.S. would like to acknowledge the Interdisciplinary Centre for Mathematical and Computational Modeling (ICM) and Wroclaw Center of Networking and Supercomputing (WCSS) for providing the access to supercomputer facilities (grant nr. G56-4).”

