# Supplementary material for: TiO2-catalyzed synthesis of sugars from formaldehyde in extraterrestrial impacts on the early Earth
Source: Sci Rep. 2016 Mar 16;6:23199. doi: 10.1038/srep23199 (PMC4793247; doi:10.1038/srep23199)
Supplement: Supplementary Information [file srep23199-s1.pdf]

Supplementary Information to the paper:

TiO_2-catalyzed synthesis of sugars from formaldehyde in extraterrestrial impacts on the early Earth

Svatopluk Civiš,[a, †] Rafał Szabla,[b, †] Bartłomiej M. Szyja,[c,d] Daniel Smykowski,[d]
Ondřej Ivanek,[a] Antonín Knížek,[a] Petr Kubelík,[a,f]
Jiří Šponer,[b,e] Martin Ferus,[a,*] and Judit E. Šponer[b,e] *

[a] *J. Heyrovský Institute of Physical Chemistry, Academy of Sciences of the Czech Republic, Dolejškova 3, CZ−182 23 Prague 8, Czech Republic*

[b] *Institute of Biophysics, Academy of Sciences of the Czech Republic, Královopolská 135, CZ−612 65 Brno, Czech Republic*

[c] *Inorganic Materials Chemistry, Department of Chemical Engineering and Chemistry, Eindhoven University of Technology, Den Dolech 2, 5612AZ Eindhoven, The Netherlands*

[d] *Division of Fuels Chemistry and Technology, Faculty of Chemistry, Wrocław University of Technology, Gdańska 7/9, 50-344 Wrocław, Poland*

[e] *CEITEC − Central European Institute of Technology, Masaryk University, Campus Bohunice, Kamenice 5, CZ−62500 Brno, Czech Republic*

[f] *Institute of Physics, Czech Academy of Sciences, Na Slovance 2, 182 21 Prague 8, Czech Republic*

[*] *Corresponding authors: Martin Ferus, Ph.D., E−mail:* martin.ferus@jh−inst.cas.cz, *Tel.: +420 26605 3685.*
Judit E. Sponer, Ph.D., E−mail: judit@ncbr.muni.cz, *Tel.: +420 54151 7246.*

[†] *The first two authors contributed equally to the work.*

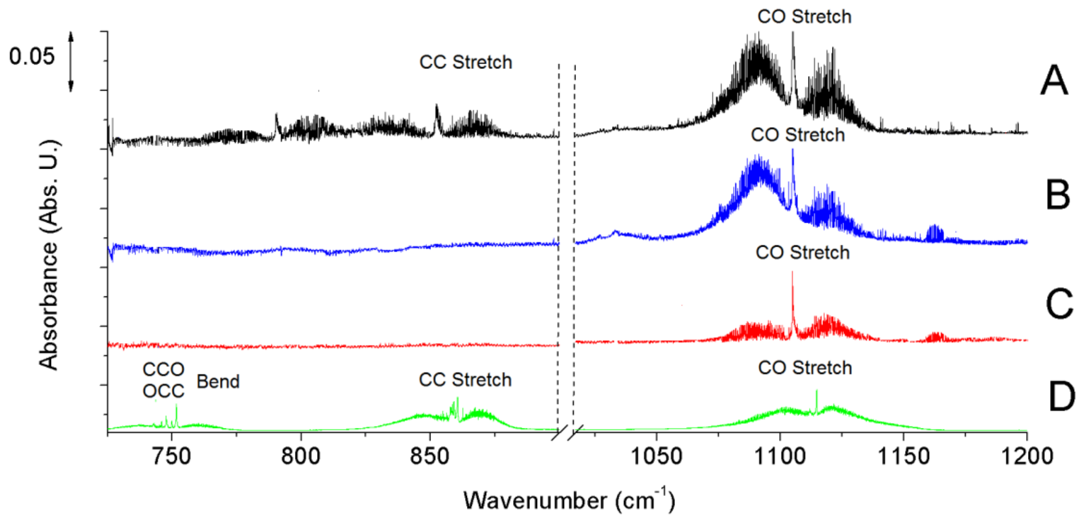

Figure S1. High−resolution infrared spectrum of (A) the volatile products formed from an icy mixture of paraformaldehyde and water in a LIDB−plasma in the presence of TiO_2, (B) the same mixture treated in a LIDB−plasma in the absence of a TiO_2−catalyst, (C) a paraformaldehyde−water mixture in vapor phase, (D) a glycolaldehyde−water mixture in vapor phase. LIBD = Laser Induced Dielectric Breakdown

Detailed description of the formaldehyde to glycolaldehyde dimerization reaction mechanism.

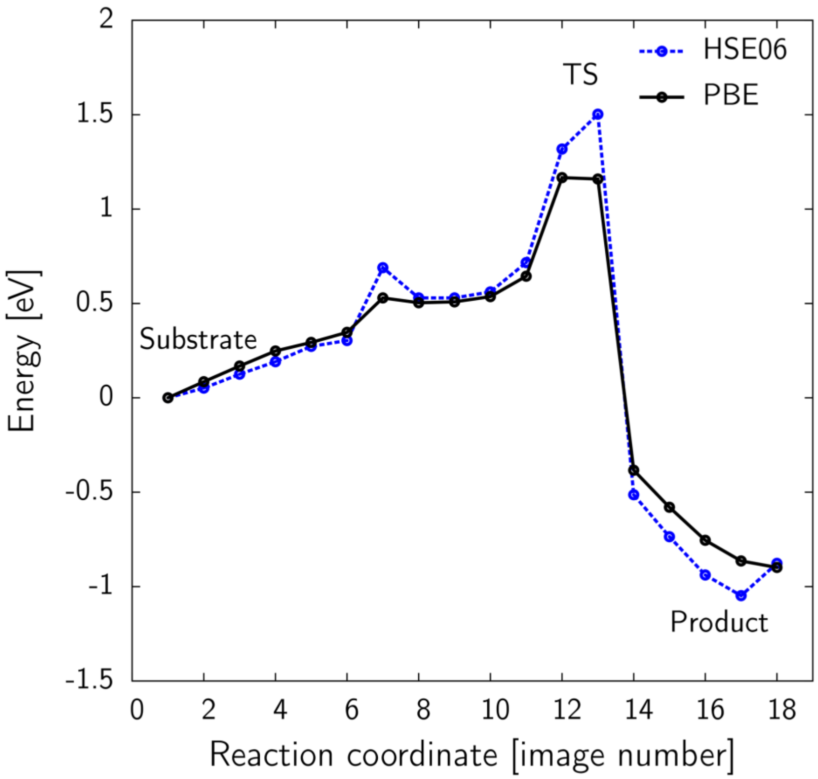

Figure S2. Relative energies along the minimum energy path (MEP) leading to the formation of glycolaldehyde from two formaldehyde molecules adsorbed at the defect site of the TiO_2-anatase (001) surface.

Figure S2 presents the minimum energy path optimized at the PBE (Perdew-Burke-Ernzerhof)[1] level of theory, using the nudged elastic band (NEB) method.[2] The whole reaction can be divided into two stages first of which refers to the formation of the C-C bond between two formaldehyde molecules. Since the corresponding intermediate lies in a very shallow plateau region of the potential energy surface, we were unable to optimize it (i.e. the optimizations converged back to the substrates). The second stage of the reaction corresponds to hydrogen atom transfer between the two reacting formaldehyde molecules and results in the formation of a hydroxyl group. The overall energy barrier of approximately 1.2 eV (~116 kJ * mol^{-1}) should be accessible at standard ambient temperature and pressure. Furthermore, the high temperature of the reaction mixture generated during the meteoritic impact could additionally promote this process.

It was suggested that GGA functionals (like PBE) do not offer a satisfactory description of the character of O-vacancies in TiO_2 due to insufficient cancellation of the self-interaction error.[3] Therefore, we benchmarked the performance of the PBE functional against a higher level

method. For that purpose we performed single point energy calculations with the HSE06 range-separated functional[4] based on the PBE-optimized geometries obtained from the preceding NEB calculation (see Figure S2). The HSE06 functional was shown to improve the theoretical description of oxygen vacancies in TiO_2 and correct positioning of energy levels when compared to experiments.[5] It is apparent from Figure S2 that both functionals yield qualitatively consistent description of the potential energy surface along the reaction path. The quantitative deviations are most pronounced in the near proximity of saddle points, and indicate slightly higher energy barriers for the two reaction steps. It is important to note, that the HSE06 energy barriers were obtained without geometry relaxation and a full NEB calculation at the HSE06 would yield somewhat lower values. Such a calculation is currently not feasible computationally. Nonetheless, the results are sufficient to conclude that the PBE functional provides a reliable qualitative picture of the reaction mechanism and good estimates of the relative energies along the reaction path.

Methods

Measurement of the high−resolution infrared spectrum of irradiated samples and vapor−phase standards. Glass irradiation cell equipped with a Pyrex window of 10 cm diameter has been filled with 1 g of paraformaldehyde (reagent grade, crystalline, CAS 30525−89−4, Sigma Aldrich), 1 ml of deionized water and 0.1 g of anatase TiO_2 (99.8%, powder, CAS 1317−70−0, Sigma Aldrich), and 1 atm of inert nitrogen gas. The sample has been subsequently frozen by liquid nitrogen, transferred to the Prague Asterix Laser System facility (PALS) and irradiated by 10 laser pulses of 150 J in energy (time interval ≈ 350 ps, wavelength of 1.315 μm, output of 428 MW). The laser beam has been focused using CaF_2 lens to achieve an output density approximately of $10^{14} - 10^{16}$ W/cm^2. The experiment mimics the high−density energy plasma in an asteroid impact (plasma temperature of 4500 K, shock wave, emission of hard UV and XUV radiation).

Prior to spectroscopic measurements the frozen samples were melted in vacuum. High resolution Fourier transform infrared (HR−FTIR) spectra of the vapor phase were measured in a multipass White cell reaching an optical path of 35 m. The cell was interfaced to a sealable glass vacuum line used for the transfer of the vapors formed upon the evaporation of the icy reaction mixture from the irradiation cell.

The spectrometer Bruker IFS 125 HR was subsequently evacuated and operated in the measurement mode from 650 − 5500 cm^{-1} using a HgCdTe nitrogen cooled detector and a KBr beamsplitter. 100 scans were acquired with 40 kHz scanning mirror speed with a resolution of 0.02 cm^{-1}. The measured interferograms were apodised with the Blackmann−Harris apodisation function.

For comparison, we have also recorded the spectrum of a sample, which was prepared in the same way as described above, but did not contain anatase TiO_2. To identify the products formed upon the simulated high−density energy event, we have also recorded the vapor−phase spectra of paraformaldehyde (reagent grade, crystalline, CAS 30525−89−4, Sigma Aldrich), glycolaldehyde (crystalline dimer, mixture of stereoisomers, CAS 23147−58−2, Sigma Aldrich) and glyceraldehyde (DL mixture, assay ≥90%, CAS 56−82−6 Sigma Aldrich) − water mixtures. 1 g of powdered samples has been mixed with deionized water in a vessel, subsequently frozen by liquid nitrogen and evacuated. The frozen samples have been melted again and they have been evaporated under continuous stream of inert nitrogen gas (5 Torr) to the multipass cell in the temperature range from 50 ^{o}C up to 130 ^{o}C. 100 scans were recorded to acquire the spectra during the evaporation procedure.

GC−MS analysis of the non−volatile fraction of the products formed upon irradiation with a high−power laser. The irradiated and melted samples were evaporated under vacuum inside a vial vessel and analyzed for the presence of saccharides. The measurements were performed using a ITQ 1100 GC−Ion Trap MS system (ThermoScientific, USA), equipped with an Xcalibur MS Platform using a non−polarTG−SQC column (ThermoScientific, USA). 17 μL of hexamethyldisilazane (99% HMDS, CAS 999−97−3, Sigma Aldrich), 6μL of chlorotrimethylsilane (99% TMCS, CAS 75−77−4, Sigma Aldrich), and 52 μL of pyridine (99.5% anhydrous, Scharlau) were added to the residue as derivatization agents and aprotic

solvent, respectively. The vial was then heated at 70 °C for two hours. Subsequently, 0.5μL of the sample was injected into the chromatograph, and the measurements were performed using a column temperature range of 180−280 °C with a temperature gradient of 30 °C min^{-1}. The mass spectrum was compared with the GC chromatograms and MS spectra of D−forms of ribose, lyxose, xylose (99%), arabinose (98%), threose (60% syrup), ribulose (1M solution) and xylulose and xylose (98% syrup) standards (all from Sigma Aldrich). Liquid−phase standards (i.e. threose, ribulose and xylulose) were evaporated under vacuum in the presence of phosporus pentoxide prior to GC−MS analysis. Chromatograms of the irradiated samples are depicted in Figure S3. Identification was performed by a comparison of retention times and mass spectra of the standard. Data (in comparison with those taken from the NIST-library) are supplied in Figures S4, S5, S6 and S7. Statistical treatment of comparative analysis is supplied in Table S1. Measurement of standards is crucial in our case, because the NIST library is based on quadruple analyzer, while our ITQ 1100 spectrometer is equipped with ion trap. Therefore, the NIST probability is very low even for standards (8.6 – 26.3 %). Also, existence of a wide range of conformers and isomers significantly influences the analysis. Moreover, identification of such complicated group of analytes as sugars must be based on analysis of real samples retention times. On the other hand, R. Match factor[6] reaches reasonable levels between 758 – 687 units. This factor is derived from a modified cosine of the angle between the spectra (normalized dot product) assuming each peak as a single point in a multidimensional hyperspace defined by the m/z variables. Its value may be regarded as the inverse of distance of the two point representations when each spectral vector has unit length. Also, retention times of individual sugars are in good agreement with standard measurement.

Table S1. Statistical treatment of the sugars identification.

Compound	Sample	Position	R.Match	NIST Probability (%)	Retention Time (min)
Threose, tris(trimethylsilyl)-	Standard	#1	854	23.4	3.01
	Sample	#2	718	10.1	3.01
Arabinopyranose, tetrakis-O-(trimethylsilyl)-	Standard	#1	886	8.08	3.30
	Sample	#4	686	2.68	3.31
Ribofuranose, tetrakis(trimethylsilyl)-	Standard	#1	758	26.3	3.70
	Sample	#4	721	3.3	3.74
Xylofuranose, 1.2.3.5-tetrakis-O-(trimethylsilyl)-	Standard	#1	886	8.63	3.98
	Sample	#9	687	1.44	3.97

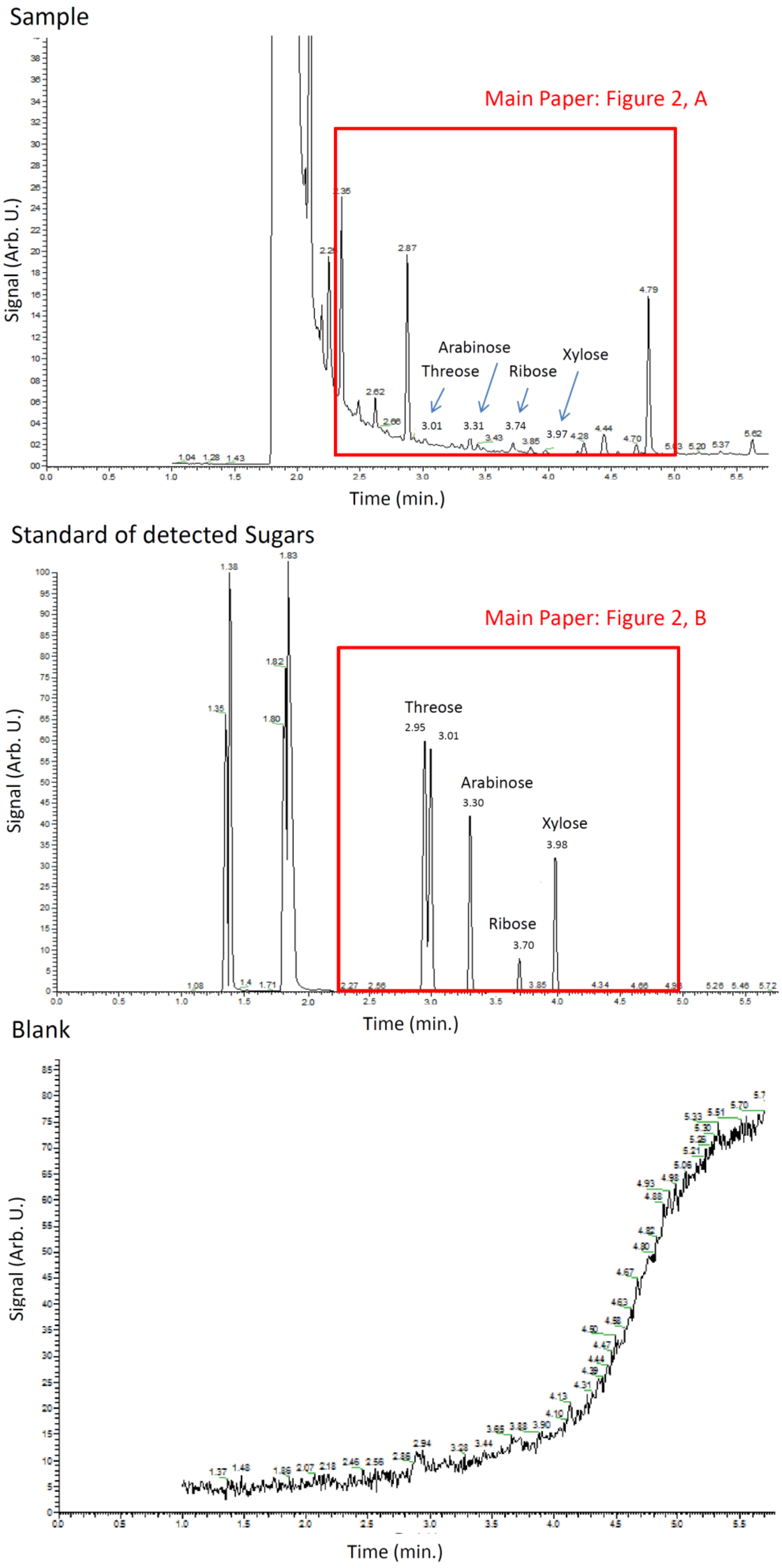

Figure S3. Chromatogram of the sample irradiated with 10 laser pulses along with the measurement of the standards of all the detected sugars in the range of 0 – 5.5 min. The most important retention time range used for the sugars' identification from 2.3 to 5.0 min is depicted in Figure 2 in the main text. The retention time range used for identification of glycolaldehyde, glycerol and diglycolic acid in the range of 5.0 – 10.0 min is depicted in Figure 1 of the main text.

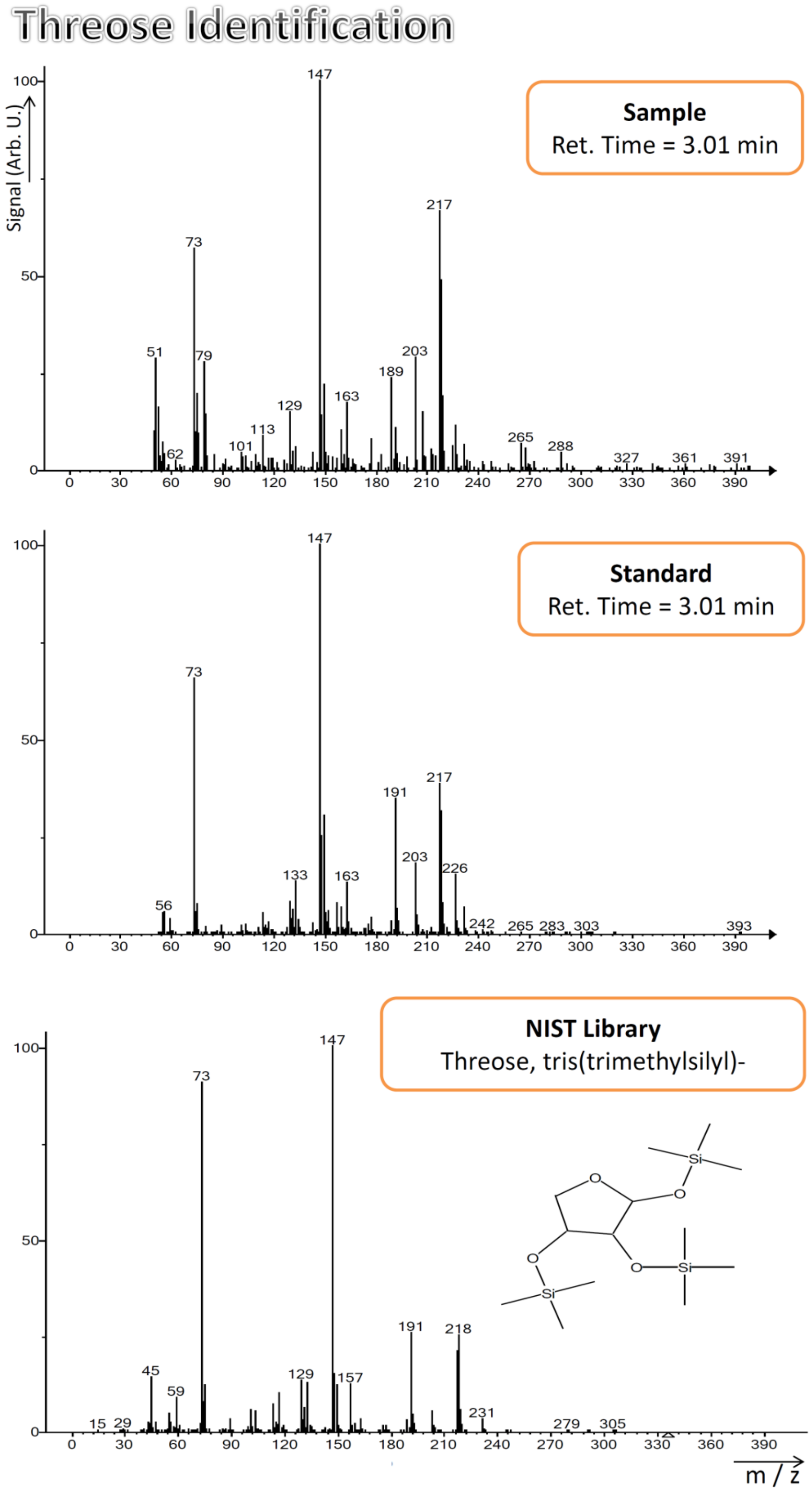

Figure S4. Comparison of the threose mass spectra at retention time position of 3.01 min in the irradiated sample, in the standard, as well as in the NIST library.

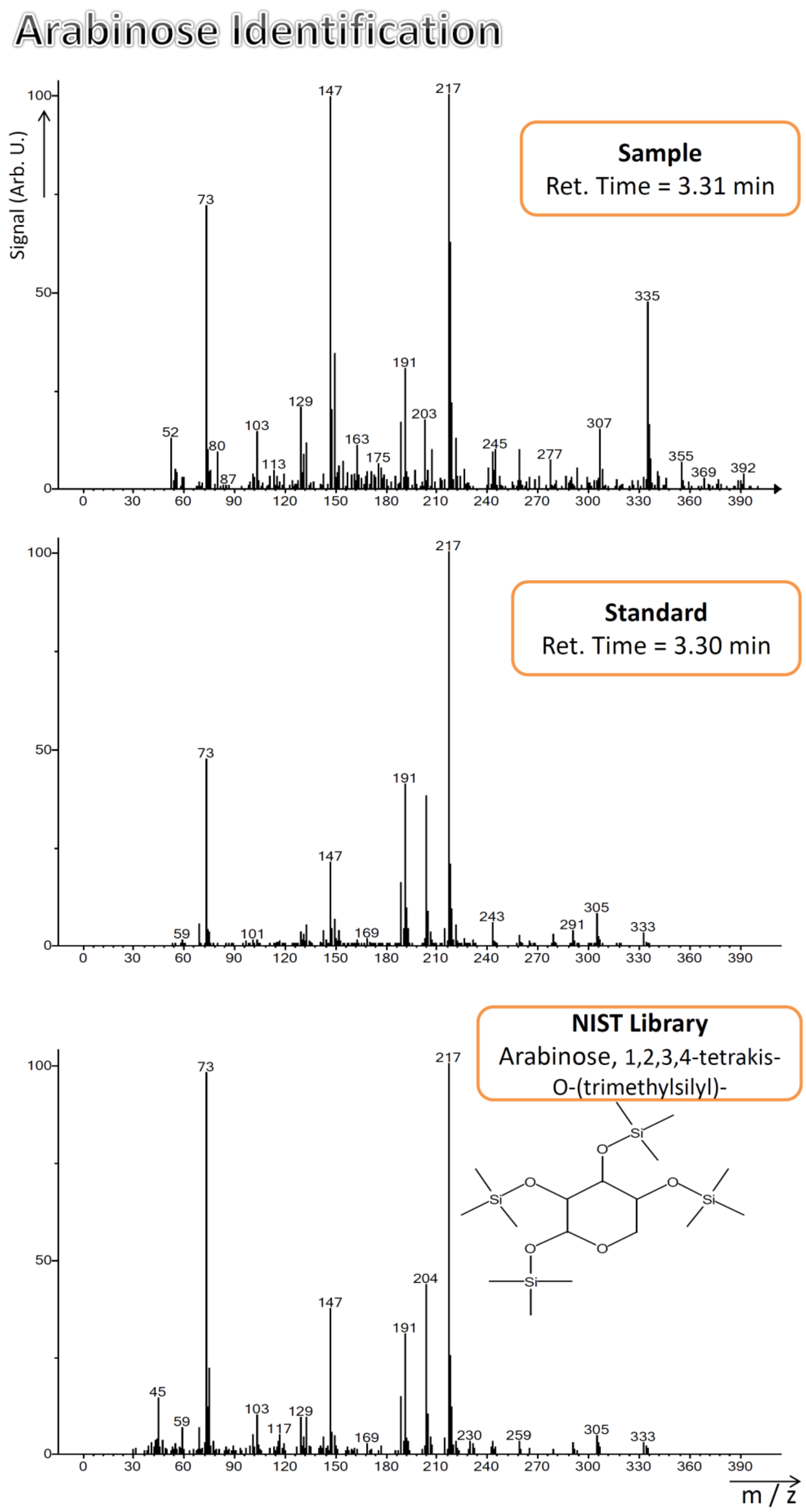

Figure S5. Comparison of the arabinose mass spectra at retention time position of 3.30 and 3.31 min in the irradiated sample, in the standard, as well as in the NIST library.

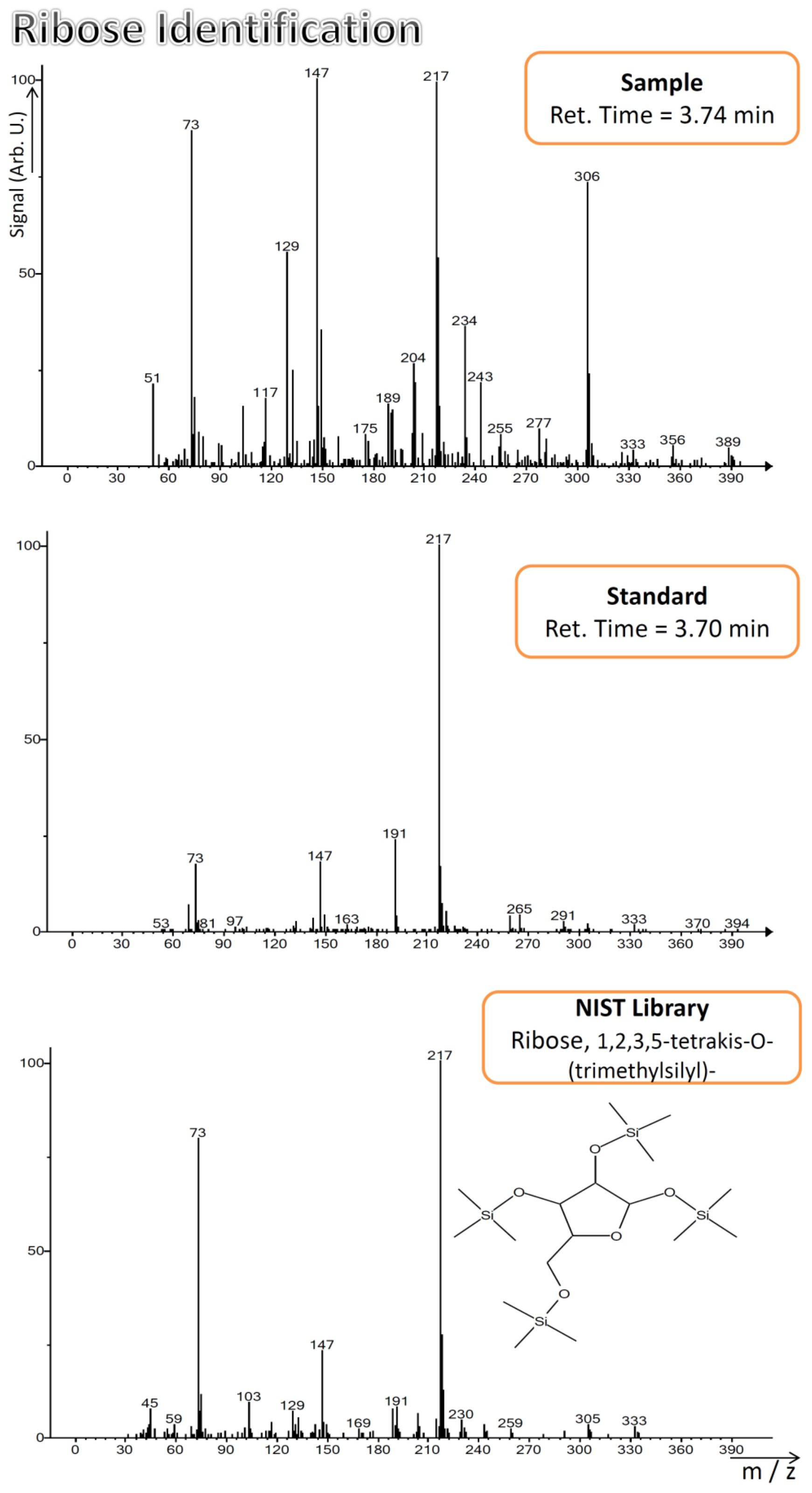

Figure S6. Comparison of the ribose mass spectra at retention time position of 3.74 and 3.70 min min in the irradiated sample, in the standard, as well as in the NIST library.

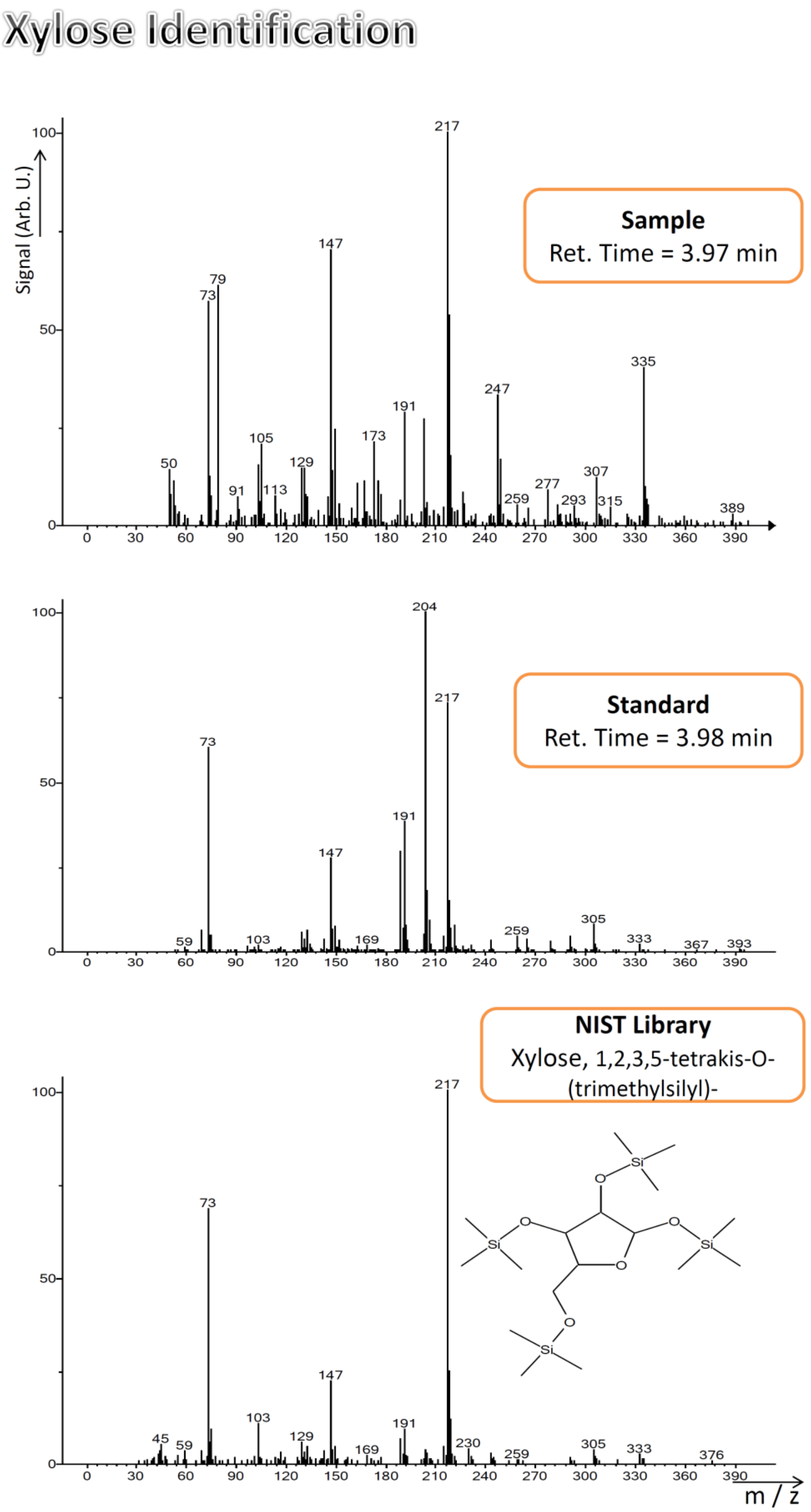

Figure S7. Comparison of the xylose mass spectra at retention time position of 3.97 and 3.98 min in the irradiated sample, in the standard, as well as in the NIST library.

References:

1. J. P. Perdew, K. Burke, M. Ernzerhof, *Phys. Rev. Lett.* **1996,** *77*, 3865–3868.
2. G. Henkelman, B. P. Uberuaga, H. Jónsson, *J. Chem. Phys*. **2000**, *113*, 9901−9904.
3. U. Aschauer, Y. He, H. Cheng, S.-Ch. Li, U. Diebold, A. Selloni, *J. Phys. Chem. C* **2010**, *114*, 1278–1284.
4. A.U. Krukau, O.A. Vydrov, A.F. Izmaylov, G.E. Scuseria, *J. Chem. Phys* **2006**, *125*, 224106.
5. Janotti, J. B. Varley, P. Rinke, N. Umezawa, G. Kresse, and C. G. Van de Walle, *Phys. Rev. B* **2010,** *81*, 085212.
6. E. Stein: NIST Standard Reference Database 1A NIST/EPA/NIH Mass Spectral Library (NIST 08) and NIST Mass Spectral Search Program (Version 2.0f), User's Guide. The NIST Mass Spectrometry Data Center.
